# Anti-apoptotic role of the sonic hedgehog signaling pathway in the proliferation of ameloblastoma

**DOI:** 10.3892/ijo.2013.2010

**Published:** 2013-07-08

**Authors:** SHIORI KANDA, TAKESHI MITSUYASU, YU NAKAO, SHINTARO KAWANO, YUICHI GOTO, RYOTA MATSUBARA, SEIJI NAKAMURA

**Affiliations:** Section of Oral and Maxillofacial Oncology, Division of Maxillofacial Diagnostic and Surgical Sciences, Faculty of Dental Science, Kyushu University, Higashi-ku, Fukuoka 812-8582, Japan

**Keywords:** ameloblastoma, sonic hedgehog signaling, apoptosis, proliferation, AM-1, neutralizing antibody, cyclopamine, odontogenic tumor

## Abstract

Sonic hedgehog (SHH) signaling pathway is crucial to growth and patterning during organogenesis. Aberrant activation of the SHH signaling pathway can result in tumor formation. We examined the expression of SHH signaling molecules and investigated the involvement of the SHH pathway in the proliferation of ameloblastoma, the most common benign tumor of the jaws. We used immunohistochemistry on ameloblastoma specimens and immunocytochemistry and reverse transcription-PCR on the ameloblastoma cell line AM-1. We also used the inhibitors of SHH signaling, SHH neutralizing antibody and cyclopamine, to assess the effects of SHH on the proliferation of AM-1 cells. We detected expression of SHH, patched, GLI1, GLI2 and GLI3 in the ameloblastoma specimens and AM-1 cells. The proliferation of these cells was significantly inhibited in the presence of SHH neutralizing antibody or cyclopamine; this was confirmed by BrdU incorporation assays. Furthermore, in the presence of SHH neutralizing antibody, nuclear translocation of GLI1 and GLI2 was abolished, apoptosis was induced, BCL-2 expression decreased and BAX expression increased. Our results suggest that the SHH signaling pathway is constitutively active in ameloblastoma and plays an anti-apoptotic role in the proliferation of ameloblastoma cells through autocrine loop stimulation.

## Introduction

Ameloblastoma, the most common benign tumor of the jaws, is characterized by slow growth and local invasion with potentially destructive behavior. It is thought to arise from the odontogenic epithelium because of a histological resemblance to the enamel organ of the developing tooth germ. However, the detailed mechanisms of its proliferation and invasion are not understood.

Sonic hedgehog (SHH) signaling pathway is crucial to growth and patterning during organogenesis including the limb bud, hair, glands, gut and gonads (1–3). SHH is a secreted protein that activates a membrane-receptor complex formed by patched (PTCH) and smoothend (SMO) (1,4). PTCH and SMO are membrane-bound proteins with seven transmembrane domains, respectively (5,6). In the absence of SHH, PTCH inhibits SMO, whereas the binding of SHH to PTCH suspends this inhibition, thereby activating zinc finger DNA-binding proteins GLI1, GLI2 and GLI3 (1). The GLI proteins mediate SHH signaling by translocating from the cytoplasm to the nucleus to act as transcription factors to activate target genes (1,7,8). Recent studies have implicated inherited or sporadic alterations in SHH signaling pathway genes in a number of developmental defects and aberrant activation of the SHH signaling pathway can result in tumor formation (9–16). In the developing tooth germ, SHH is expressed in the epithelial component and regulates the proliferation and differentiation of ameloblasts (17,18). Furthermore, alterations in PTCH expression have been demonstrated in keratocystic odontogenic tumors, which are characterized by cystic structures with proliferation of the odontogenic epithelium within the jaw (19,20). These studies suggest that the SHH signaling pathway is closely associated with proliferation of odontogenic epithelial cells. In this study, we examined the expression of SHH, PTCH and GLI proteins and elucidated the functional roles of the SHH signaling pathway, in the proliferation of ameloblastoma.

## Materials and methods

### Patients

The subjects gave informed consent before enrolment. Specimens were surgically removed from 29 patients with primary ameloblastoma (male, 22 and female, 7 cases, mean age, 37.8±19.4 years; age range, 14–80 years) at the Department of Oral and Maxillofacial Surgery, Kyushu University Hospital. Following the initial biopsy, all specimens were fixed in 4% buffered formalin solution and embedded in paraffin blocks. Subsequently, the specimens were processed into 5-μm thick sections and stained with hematoxylin and eosin. The tumors were further classified as 17 follicular and 12 plexiform types according to the World Health Organization guidelines for histologic typing of odontogenic tumors (21).

### Immunohistochemistry

The sections were deparaffinized in xylene and hydrated in graded ethanol. For antigen retrieval, the sections were immersed in Target Retrieval Solution (Dako, Denmark) and autoclaved at 121°C for 5 min. After elimination of endogenous peroxide activity and blocking by incubation with 10% normal goat serum (Nichirei Bioscience, Japan), the sections were incubated with primary antibody overnight. The following antibodies were used: anti-human SHH monoclonal (Abcam, UK; diluted 1:100), anti-human PTCH polyclonal (Santa Cruz, USA; diluted 1:100), anti-human GLI1 polyclonal (Abcam; diluted 1:80), anti-human GLI2 polyclonal (Abcam; 1:200) and anti-human GLI3 polyclonal (Novus Biological, USA; 1:100) antibodies. The sections were then incubated with horseradish peroxidase-conjugated secondary antibodies for 1 h. The immunoreactivities were visualized by immersing the sections in 3,3′-diaminobenzidine (Nichirei Bioscience). Subsequently, the sections were dehydrated, cleared with xylene and finally mounted. Negative controls were prepared by substituting phosphate-buffered saline for primary antibody.

### Cell culture and immunocytochemistry

The human ameloblastoma cell line AM-1, which was established from human ameloblastoma tissue and immortalized by the transfection of human papillomavirus type 16 DNA, was maintained in defined keratinocyte-serum-free medium supplemented with adjunctive growth supplement (22). The immortalized human keratinocyte cell line (HaCat) was cultured in Dulbecco’s modified Eagle’s medium/F-12 (Sigma-Aldrich, USA) supplemented with 10% fetal bovine serum. All cell lines were maintained with 100 U/ml penicillin/streptomycin in a humidified atmosphere of 5% CO_2_ at 37°C.

For immunocytochemistry, cultured cells were fixed in 75% methanol and then incubated with primary antibodies as described above and the anti-human BCL-2 polyclonal (Ana Spec, USA; 1:500) and anti-human BAX polyclonal (R&D Systems, USA; 1:500). Subsequently, the cells were incubated with Alexa Fluor^®^ 488- or 546-conjugated secondary antibodies (Molecular Probes, USA; diluted 1:400). The cells were counterstained with 1 μg/ml Hoechst 33342 (Molecular Probes) and observed under a fluorescence microscope (BZ-8000; Keyence, Japan).

### RNA extraction and cDNA synthesis

Total RNA was extracted from cultured cells using a PureLink™ RNA Mini kit (Invitrogen, USA). The amount of RNA extracted from each sample was measured spectrophotometrically on a NanoDrop 1000 (Thermo Scientific, USA). Of the total RNA preparation 2 μg was used for cDNA synthesis. Briefly, RNA was incubated for 15 min at 42°C with 25 U/μl of recombinant RNase inhibitor (Nacalai Tesque, Japan), 1.0 μl of 50 μM random hexamers (Applied Biosystems), 2.0 μl of each 2.0 μM dNTP (Toyobo, Japan) and 50 U/μl of Moloney murine leukemia virus reverse transcriptase (Roche Diagnostics, Switzerland).

### Reverse transcription-PCR (RT-PCR)

For RT-PCR, 100 ng of template DNA, 0.5 μl of 20 pM sense and antisense primers, 1.0 μl of 25 mM MgCl_2_, 1.25 μl of 10X Taq DNA polymerase buffer (Bio Basic, Canada), 5 U/μl of Taq DNA polymerase (Bio Basic), 0.5 μl of 2.0 mM dNTP mix (Toyobo) and 9.65 μl of sterilized water were used in a total volume of 13.5 μl. The PCR conditions were: 25 cycles of denaturing at 94°C for 30 sec, annealing at 60°C for 30 sec and elongation at 72°C for 15 sec. For amplification of specific regions of target genes, the primers used were: *SHH*, forward 5′-GATGACTCAGAG GTGTAAGGACAA-3′ and reverse 5′-CCACCGAGTTCTCT GCTTTCA-3′; *PTCH*, forward 5′-GGATCATTGTGATGGTC CTG-3′ and reverse 5′-GTCAGAAAGGCCAAAGCAAC-3′; *GLI1*, forward 5′-CACCACATCAACAGCGAGCA-3′ and reverse 5′-TTCCGGCACCCTTCAAACG-3′; *GLI2*, forward 5′-AGCAGCAGCAACTGTCTGAGTGA-3′ and reverse 5′-GAC CTTGCTGCGCTTGTGAA-3′; *GLI3*, forward 5′-TCCAAC ACAGAGGCCTATTCCAG-3′ and reverse 5′-CTCTTGTTGT GCATCGGGTCA-3′; glyceraldehyde 3-phosphate dehydrogenase (*GAPDH*), forward 5′-ATCAGCAATGCCTCCT GCA-3′ and reverse 5′-ATGGCATGGACTGTGGTCAT-3′. The housekeeping gene *GAPDH* was used as the internal control.

### Water-soluble terazolium (WST)-8 cell proliferation assay

Cell proliferation assays were performed using the WST-8 Cell Counting kit (Dojin, Japan), according to the manufacturer’s instructions. Briefly, 3.0×10^3^ cells/well were seeded into 96-well microtiter plates. After 24-h incubation, an inhibitor of sonic hedgehog signaling-SHH neutralizing antibody (1 ng/ml; StemRD, USA) or cyclopamine (1 mM; Enzo Life Science, USA) was added to each well and the absorbance at 450 nm was measured using a microplate reader (Multiskan FC, Thermo Scientific).

### Apoptosis assay

The Annexin V assay was performed to detect apoptotic cells. Briefly, 3.0×10^4^ AM-1 cells were seeded into culture plates, after 24-h incubation, 1 ng/ml SHH neutralizing antibody was added to each well and further incubated for 48 h. Apoptotic cells were stained by Annexin V conjugated with fluorescein isothiocyanate (MBL, Japan) and counted under a fluorescence microscope.

### Statistical analyses

All statistical analyses were performed using JMP software version 8 (SAS Institute, Japan).

## Results

### Expression of SHH molecules in ameloblastoma and normal gingiva

**Figure 1:**
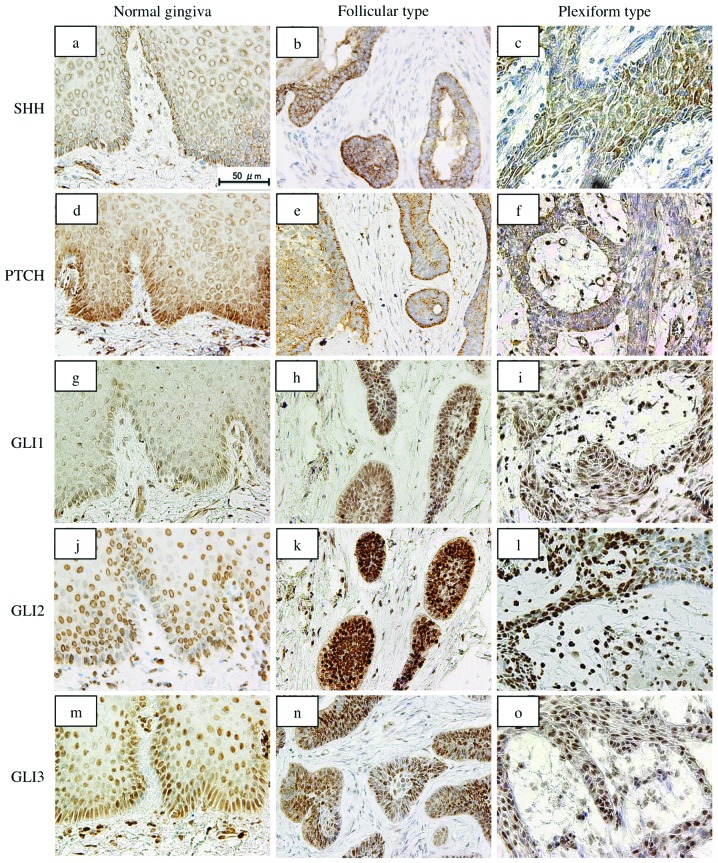
Immunohistochemical staining of SHH, PTCH, GLI1, GLI2 and GLI3 in ameloblastoma specimens. In the normal gingiva, SHH is expressed strongly in the cytoplasm of basal cells (a). The expression of PTCH is observed in the cell membrane and cytoplasm of the epithelial cells (d). GLI1 (g), GLI2 (j) and GLI3 (m) localize in the nucleus of the epithelial cells. In ameloblastoma, immunoreactivity for SHH, PTCH, GLI1, GLI2 and GLI3 is seen in almost all the tumor cells. SHH is expressed in the cytoplasm (b and c), PTCH in the cytoplasm and cell membrane (e and f) and GLI proteins only in the nucleus (h, i, k, l, n and o). The reactivity is stronger in the peripheral cuboidal and columnar cells than in the central polyhedral cells. Bar, 50 μm.

In the normal gingiva, immunoreactivity for SHH, PTCH, GLI1, GLI2 and GLI3 was more evident in the epithelial cells than in the stromal cells. SHH was strongly expressed in the cytoplasm of basal cells and weakly in the cells of the stratum spinosum. The expression of PTCH was observed in the cell membrane and cytoplasm of the epithelial cells. GLI1, GLI2 and GLI3 were localized in the nucleus of the epithelial cells. GLI1 and GLI3 were mainly expressed in the basal layer, while GLI2 was strongly expressed in the parabasal cells rather than basal cells. In ameloblastoma, immunoreactivity for SHH, PTCH, GLI1, GLI2 and GLI3 was seen in almost all tumor cells, but not in the stromal cells. SHH was expressed in the cytoplasm, PTCH in the cytoplasm and cell membrane and the GLI proteins only in the nucleus. The reactivity was stronger in the peripheral cuboidal and columnar cells than in the central polyhedral cells of the tumor nests. There was no difference in the expression pattern of these proteins between the follicular and plexiform types (Fig. 1).

### Expression of SHH-related genes and gene products in the ameloblastoma cell line AM-1

**Figure 2:**
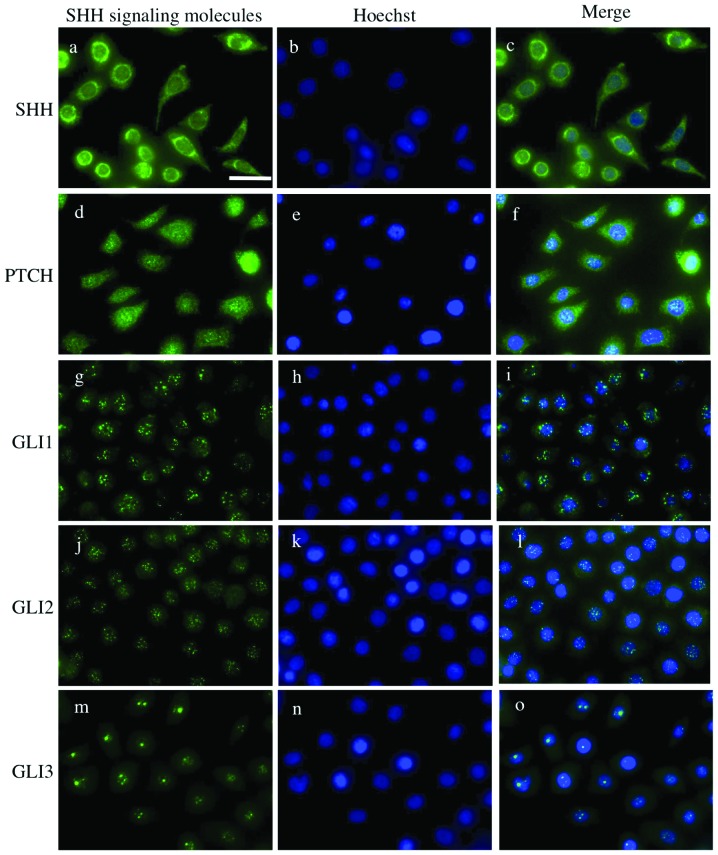
Immunocytochemical staining of SHH, PTCH, GLI1, GLI2 and GLI3 in AM-1 cells. (a–c) Expression of SHH is detected mainly in the cytoplasm. (d–f) Immunoreactivity for PTCH is observed in the cell membrane and cytoplasm. Expression of GLI1 (g–i), GLI2 (j–l) and GLI3 (m–o) is localized in the nucleus, but not in the cytoplasm or membrane. Bar, 20 μm.

**Figure 3:**
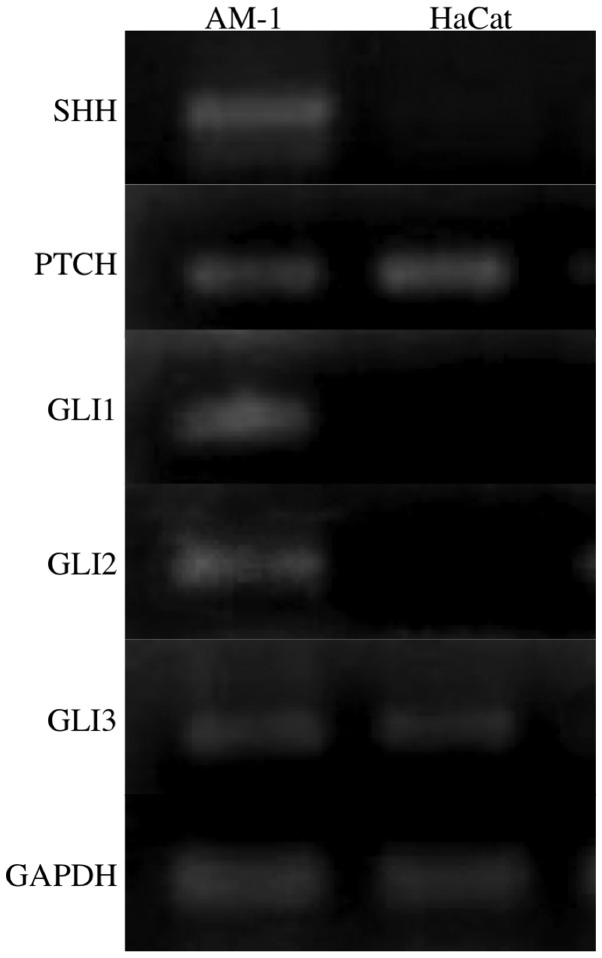
Expression of SHH signaling-associated genes in AM-1 cells by RT-PCR. *SHH, PTCH, GLI1, GLI2* and *GLI3* are expressed in AM-1 cells, while *PTCH* and *GLI3* are also expressed in HaCat cells.

By immunocytochemistry, SHH was mainly expressed in the cytoplasm. Immunoreactivity for PTCH was observed in the cell membrane and cytoplasm. The expression of GLI1, GLI2 and GLI3 was localized in the nucleus, but not in the cytoplasm or membrane (Fig. 2). RT-PCR analyses revealed that *SHH, PTCH, GLI1, GLI2* and *GLI3* were expressed in the AM-1 cells, while *PTCH* and *GLI3* were also expressed in the HaCat cells (Fig. 3).

### SHH neutralizing antibody and cyclopamine suppress AM-1 cell proliferation

**Figure 4:**
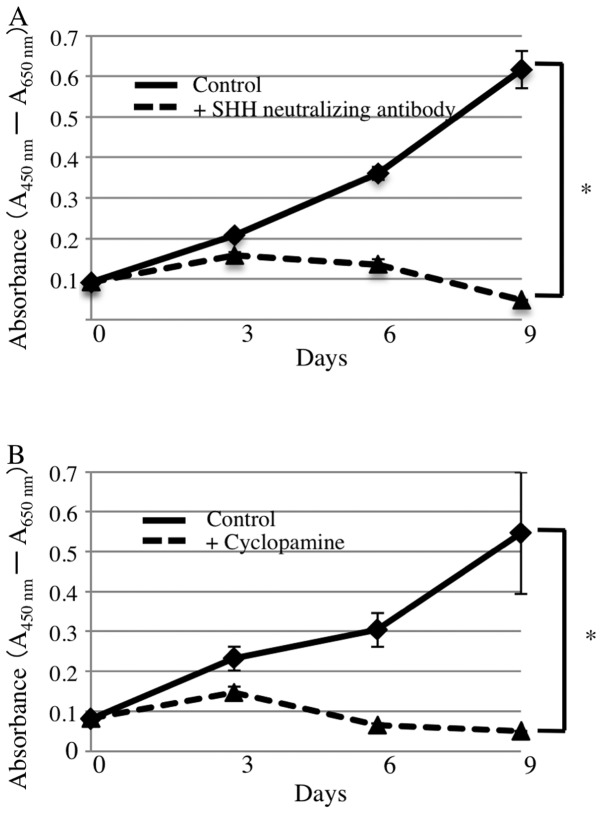
Effect of SHH neutralizing antibody or cyclopamine on AM-1 cell proliferation: WST-8 assay. (A) Cell proliferation is significantly inhibited in the presence of 1 ng/ml SHH neutralizing antibody compared with in its absence (repeated measures ANOVA, ^*^p<0.05). (B) Adding 1 mM cyclopamine also suppresses proliferation of AM-1 cells (repeated measures ANOVA, ^*^p<0.05). The data are shown as the mean ± standard deviation of 3 independent experiments.

**Figure 5:**
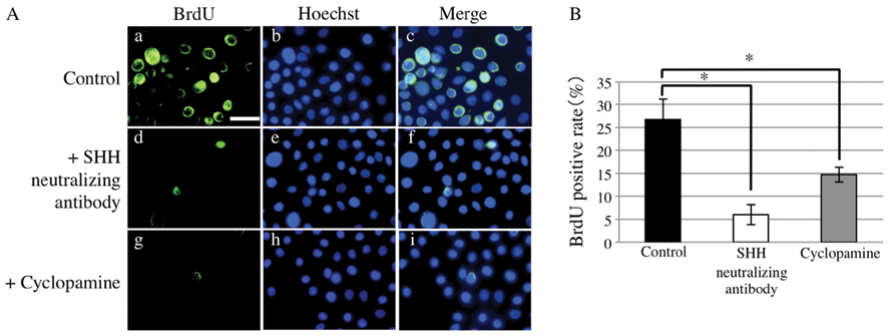
Effect of Shh neutralizing antibody or cyclopamine on AM-1 cell proliferation: BrdU positivity. (A) BrdU-positive cells in the controls (a–c) or in the presence of SHH neutralizing antibody (d–f) or cyclopamine (g–i). Bar, 20 μm. (B) The number of BrdU-positive cells is significantly lower in the presence of SHH neutralizing antibody or cyclopamine than in the controls (Mann-Whitney U test, ^*^p<0.05). The data are shown as the mean ± standard deviation of 3 independent experiments.

To examine the effects of SHH on the proliferation of AM-1 cells, we added SHH neutralizing antibody or cyclopamine, both inhibitors of SHH signaling, to the culture medium. In the WST-8 assay, cell proliferation in the presence of 1 ng/ml SHH neutralizing antibody was significantly inhibited compared with that of the control (repeated measures analysis of variance (ANOVA), p<0.05) (Fig. 4A). The addition of 1 mM cyclopamine also suppressed proliferation of AM-1 cells (repeated measures ANOVA, p<0.05) (Fig. 4B). BrdU incorporation assays revealed that the BrdU positivity rate in the presence of Shh neutralizing antibody or cyclopamine was significantly lower than that in the controls (Mann-Whitney U test, p<0.05) (Fig. 5).

### Nuclear translocation of Gli proteins is abolished by SHH neutralizing antibody

**Figure 6:**
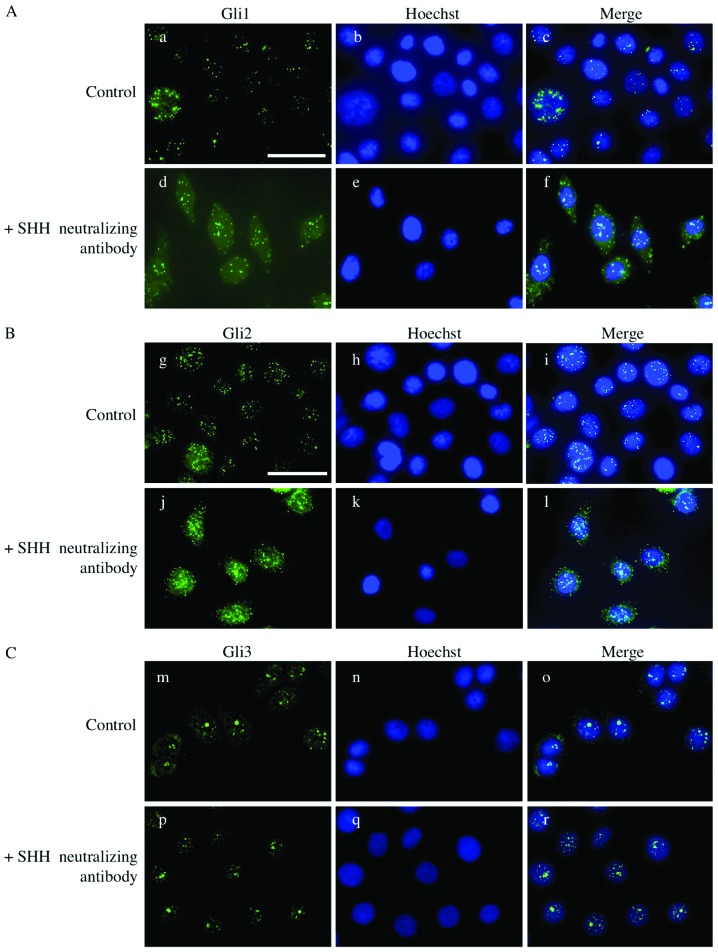
Effect of SHH neutralizing antibody on nuclear translocation of GLI1, GLI2 and GLI3 in AM-1 cells. In the control groups, immunoreactivity for GLI1 (a–c), GLI2 (g–i) and GLI3 (m–o) is observed in the nucleus of AM-1 cells. However, in the presence of SHH neutralizing antibody, GLI1 (d–f) and GLI2 (j–l) are detected in the cytoplasm rather than the nucleus. GLI3 (p–r) remains in the nucleus. Bars, 20 μm.

To examine whether SHH signal transduction is affected by SHH neutralizing antibody, we performed immunocytochemical staining for GLI proteins. In the control groups, immunoreactivity for GLI1, GLI2 and GLI3 was observed in the nucleus of AM-1 cells. However, in the presence of SHH neutralizing antibody, the expression of GLI1 and GLI2 was detected in the cytoplasm rather than the nucleus suggesting that the nuclear translocation of GLI proteins is abolished by SHH neutralizing antibody. GLI3 remained in the nucleus after the addition of SHH neutralizing antibody (Fig. 6).

### SHH neutralizing antibody induces apoptosis of AM-1 cells

**Figure 7:**
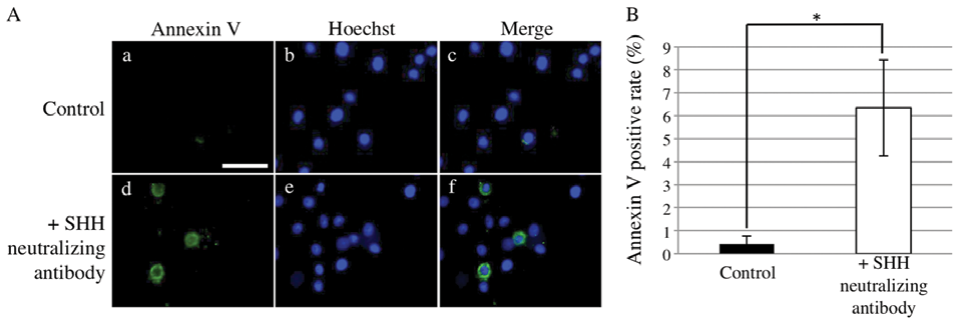
Effect of SHH neutralizing antibody on AM-1 cell apoptosis. (A) Detection of apoptotic AM-1 cells by Annexin V staining (a–f). Bar, 20 μm. (B) Annexin V-positive cells are significantly more common in the presence of SHH neutralizing antibody than in its absence (Mann-Whitney U test, ^*^p<0.05).

**Figure 8:**
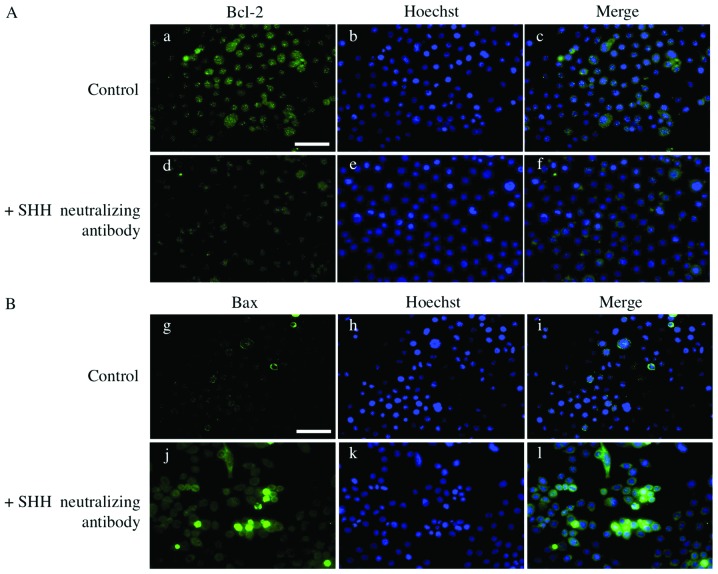
Expression of BCL-2 and BAX in AM-1 cells with or without SHH neutralizing antibody. Immunocytochemistry for BCL-2 (A) and BAX (B) in AM-1 cells. In the presence of SHH neutralizing antibody, the expression of BCL-2 is decreased, while the expression of BAX is increased. Bars, 20 μm.

We next examined the influence of SHH neutralizing antibody on the apoptosis of AM-1 cells. Annexin V-positive rates were significantly higher in the presence of SHH neutralizing antibody than those in its absence (Mann-Whitney U test, p<0.05) (Fig. 7). To examine the expression of apoptosis-associated proteins, we performed immunocytochemistry in AM-1 cells for BCL-2, an anti-apoptotic protein and BAX, a pro-apoptotic protein. The expression of BCL-2 decreased in the presence of SHH neutralizing antibody, while the expression of BAX increased (Fig. 8).

## Discussion

In the present study, we examined the expression of SHH, PTCH, GLI1, GLI2, and GLI3 in ameloblastoma and investigated their functions using an ameloblastoma cell line. Several studies have revealed that SHH signaling-associated molecules are expressed in odontogenic tumors including ameloblastoma (23–25). Kumamoto *et al* and Zhang *et al* demonstrated by immunohistochemistry that SHH, PTCH, SMO and GLI1 were expressed in all cases of ameloblastoma and that reactivity was stronger in peripheral cuboidal cells than in central polyhedral cells (23,24). Our findings were almost identical to these.

Little is known about the significance and function of SHH signaling in ameloblastoma. In a previous study, we revealed that Ki-67 and proliferating cell nuclear antigen were mainly expressed in the outer layer of tumors strongly expressing SHH signaling molecules (26). Here, we examined the association of SHH signaling with the cell proliferation of ameloblastoma using AM-1 cells. We found that the AM-1 cells expressed SHH and PTCH and that proliferation was suppressed by adding SHH neutralizing antibody or cyclopamine. Furthermore, the nuclear translocation of GLI1 and GLI2 was abolished by SHH neutralizing antibody. These results suggest that the SHH signaling pathway is constitutively activated in ameloblastoma cells and that AM-1 cells proliferate by autocrine loop SHH stimulation. Constitutive activation of SHH signaling and SHH-dependent proliferation have been found in a variety of cancers including lung, esophagus, stomach and pancreas (27–30). Ameloblastoma might now be added to this list. However, we found that the nuclear translocation of GLI3 was not abolished by Shh neutralizing antibody. It has been demonstrated that inhibition of SHH signaling by cyclopamine promotes the processing of GLI3 into a shortened form that can act as a transcription factor in cultured cells and limb explant cultures (31,32). These results suggest that a dynamic interplay between the GLI signals occurs in the proliferation of ameloblastoma, although the molecular mechanisms that control such interactions are largely undefined.

We found here that SHH neutralizing antibody induced apoptosis of AM-1 cells and demonstrated decreased BCL-2 and increased BAX expression. In a previous study, we demonstrated that BCL-2, which prevents apoptosis, was mainly expressed in the outer layer of ameloblastoma cells, whereas the inner cells (stellate reticulum-like cells and squamoid cells) did not express this protein (33). This expression pattern of BCL-2 was similar to that of SHH in ameloblastoma. Furthermore, it has been demonstrated that hedgehog signaling induced apoptosis of colorectal cancer cells in the presence of cyclopamine (34). Taken together, it seems that SHH plays an anti-apoptotic role in the proliferation of ameloblastoma cells.

Recent studies have reported SHH overexpression in basal cell carcinoma and lung squamous cell carcinoma (15,35). Furthermore, transgenic mice overexpressing SHH develop various tumors, such as basal cell carcinoma, medulloblastoma and breast carcinoma (36). These results suggest a role for SHH in tumorigenesis. Our present results suggest that inhibition of SHH signaling might be a good target for a molecular treatment for ameloblastoma, although further studies are needed to understand the precise role of the SHH signaling pathway in tumor progression.
